# A Mouse Model of Interstitial Pneumonitis Induced by Murine Cytomegalovirus Infection after Allogeneic Skin Transplantation

**DOI:** 10.1155/2013/341387

**Published:** 2013-07-02

**Authors:** Dequn Ni, Haiyang Yu, Wei Zhang, Lin Gan, Jiqiang Zhao, Mingli Wang, Jason Chen

**Affiliations:** ^1^Department of Microbiology, Anhui Medical University, Hefei, Anhui 230032, China; ^2^Key Laboratory of Gene Resource Utilization for Severe Diseases, The Ministry of Education of China and Anhui Province, Hefei, Anhui 230032, China; ^3^Department of Organ Transplantation, First Affiliated Hospital, Sun Yat-Sen University, Guangzhou, Guangdong 510080, China; ^4^Department of Pathology & Cell Biology, Columbia University, New York, NY 10032, USA

## Abstract

We investigated the effect of murine cytomegalovirus (MCMV) on interstitial pneumonia in transplant recipients in an experimental skin allograft model. Skin transplantation between C57BL/6J and BALB/c mice was performed in the presence or absence of cyclosporin A treatment. Flow cytometry showed that the number of CD4^+^ and CD8^+^ cells and the level of IFN-**γ** decreased significantly in the groups treated with cyclosporin A. We either mock-infected or infected the mice with MCMV by intranasal administration and monitored pathophysiological behavior and body weight. The infected mice were sacrificed at different days postinfection for histology, immunohistochemistry, and molecular biological evaluations. Interstitial pneumonitis was observed in positive control groups as well as in experimental group that received cyclosporin A, a skin transplant, and infected with the highest dose of virus (10^5^ PFU). Transmission electronic microscopy demonstrated the presence of herpes virus particles. MCMV DNA and glycoprotein B were demonstrated in the epithelial cells of the lung tissue in those animals by *in situ *hybridization and immunohistochemistry, respectively. Our data demonstrated the establishment of a mouse model of interstitial pneumonitis via MCMV infection after allogeneic skin transplantation.

## 1. Introduction

Cytomegalovirus (CMV), a beta herpesvirus, is the major cause of birth defects, as well as a critical pathogen to immunocompromised individuals [1]. More frequent cases of organ transplantation, blood dyscrasia, and cancer have facilitated an explosive increase in the incidence of clinically active human CMV (HCMV) infection [2, 3].

HCMV typically affects multiple organs, resulting in hepatitis, pneumonitis, retinitis, colitis, and other diseases [4]. The lungs are a prominent site of CMV infection, especially acute infection, latency, and reactivation. CMV-associated interstitial pneumonia/pneumonitis (CMV-IP) has been recognized as the most fatal cause of death among the clinical symptoms of HCMV infection in immunocompromised patients, such as recipients of skin transplantation and solid organ transplants. Thus, it is a significant impediment to successful organ transplantation [5–8].

Successful animal models of HCMV infection have been difficult to develop because of the extreme species specificity exhibited by members of the CMV family [9]. Since mouse models have been used extensively to define the contribution of various components of host immunity to the control of CMV infection, murine cytomegalovirus (MCMV) has become a good microorganism for studying systemic HCMV infections [10] and could be used to model HCMV-related disease.

HCMV pneumonitis might be the consequence of a HCMV infection by a contaminated graft implanted in a HCMV-negative recipient. It might also result from HCMV reactivation in a HCMV-positive patient in response to stress and/or immune suppression associated with organ transplantation. Our current study was designed to investigate the combined effects on the lungs of stress associated with organ transplantation and cyclosporin A- (CsA-) mediated immune suppression. We aimed to establish a mouse model of MCMV-associated interstitial pneumonitis using mice receiving allogeneic skin transplants.

## 2. Materials and Methods

### 2.1. Mice

Specific pathogen-free- (SPF-) inbred female mice, including 128 recipients (BALB/c) and 20 donors (C57BL/6J) that were 6–8 weeks old weighing 15–20 g, were purchased and maintained in the pathogen-free mouse room in the Experimental Animal Center of Anhui Medical University, China. The animal protocol was reviewed and approved by the Institutional Animal and Use Committee of Anhui Medical University.

### 2.2. Viruses

The Smith strain of MCMV (a gift from Professor H. W. Virgin, School of Medicine, Washington University, USA) was routinely propagated in BALB/c mouse embryo fibroblasts (MEFs; prepared in house), maintained in Eagle's minimal essential medium (MEM; Gibco, Invitrogen Corporation, CA, USA) containing 10% fetal bovine serum (FBS; Invitrogen Corporation, Invitrogen Shanghai Office, China), 100 U/mL penicillin, and 10 *μ*g/mL streptomycin, as previously described [11]. For MCMV titration experiments, MEFs were seeded in 12-well tissue culture plates (5 × 10^4^ cells/well) 1 day before the titration assay. MCMV was serially diluted in MEM and added (0.5 mL) in duplicate onto fibroblast monolayers for 1 h at 37°C. Unadsorbed viruses were removed. Each monolayer was then covered with culture medium containing 1.5% methylcellulose and 2% FBS. After 4 days, the medium was removed. The cells were fixed with 5% formaldehyde in saline and stained with crystal violet. After washing with saline, the cultures were examined by microscopy and the number of plaques was determined. 

### 2.3. Skin Grafting

Using trunk skin from donor mice (C57BL/6J), skin allografting was performed as previously described [12]. A square graft (1 cm × 1 cm) was placed on a graft bed prepared on the flank of a BALB/c recipient mouse. The graft was covered with protective bandages for 8 days. Allograft recipients received intraperitoneal injections of cyclosporin A (CsA; Novartis Pharma, Nürnberg, Germany, 20 mg/kg/day) daily throughout the study. Rejection was considered to have occurred when grafts exhibited dark discoloration, scabbing, and necrotic degeneration. Of the 176 BALB/c mice, 80 were given skin grafts. The rest (96 BALB/c mice) were equally divided into 6 control groups: Control-A received CsA without allografts; Control-AV received CsA treatment and 1 × 10^5^ PFU MCMV each without allografts; Control-B received allograft without CsA; Control-BV received allografts and 1 × 10^5^ PFU MCMV each without CsA treatment; and Control-C received neither allografts nor CsA. Control-CV received neither allografts nor CsA treatment, but did receive 1 × 10^5^ PFU MCMV.

### 2.4. Immunosuppression of Transplanted Mice

The number of CD4^+^ and CD8^+^ T cells in the peripheral blood of transplanted mice was determined by flow cytometric analysis using Phycoerythrin- (PE-) Texas red-conjugated anti-CD4 and fluorescein isothiocyanate- (FITC-) conjugated anti-CD8 antibodies (Santa Cruz Biotechnology, CA, USA). The IFN-*γ* levels in mouse plasma collected from the transplant recipients and control animals on day 14 after CsA injection were determined using ELISA (Abcam Corporation, MA, USA).

### 2.5. Intranasal Administration of MCMV

MCMV can be spread through saliva or breast milk in mice. Therefore, its horizontal and vertical transmissions are very common [13]. Animals in this study were infected with MCMV intranasally, since the oral, intranasal, and subcutaneous routes can be the natural MCMV infection routes [14, 15].

Intranasal injection of MCMV was carried out by injecting 80 *μ*L of MEM containing 1 × 10^2^ PFU (Group A), 1 × 10^3^ PFU (Group B), 1 × 10^4^ PFU (Group C), or 1 × 10^5^ PFU (Group D) MCMV. Mice of the mock-infected group (Group E) were injected with an equal volume of MEM. Infected animals were housed in isolation apart from control animals.

### 2.6. Virus Isolation

Mice were sacrificed at 5 d, 9 d, 14 d, or 21 d postinfection, and lung tissue was collected for analysis. For virus isolation, 100 mg of lung tissue was removed aseptically, homogenized in 1 mL of MEM, and centrifuged at 1000 rpm for 20 min at 4°C. The supernatant was filtered through 0.2 *μ*m filter, and 0.2 mL of 10-fold dilutes was placed on a MEF monolayer. The cytopathic effect (CPE) of herpes virus-like cell lesions was observed daily. Some CPEs were confirmed by immunocytochemistry with anti-MCMV gB antibody.

### 2.7. Detection of MCMV DNA and RNA by PCR and RT-PCR

DNA was extracted from lung tissue using a genomic DNA isolation Kit (Takara Biotechnology, Dalian, China). RNA was extracted from fresh lung tissue by homogenization in Trizol reagent (Invitrogen, CA, USA). The extracted RNA was purified according to the directions from the manufacturer. PCR was performed as previously described [16]. Reverse transcription-PCR (RT-PCR) analysis of the MCMV RNA was performed according to the method described by Henry and Hamilton [17]. MCMV IE and M55 genes were used as the PCR amplification targets. The primers are listed in Table 1.

### 2.8. Real-Time PCR Analysis

Real-time PCR was performed using Premix Ex Taq master mix (Takara Biotechnology, Dalian, China) in a Rotor Gene 3000 Fast system. Primers and TaqMan probes are listed in Table 1. Each sample was analyzed in triplicate. The thermal cycling conditions were 95°C for 3 min, 40 cycles of 95°C for 10 s, 54°C for 10 s, and then 60°C for 30 s.

### 2.9. Transmission Electron Microscopy (TEM)

To prepare for electron microscopy, lung tissues from mice were fixed with 2.5% glutaraldehyde in Hanks' balanced salt solution (HBSS), postfixed with 10% osmium tetroxide, dehydrated, and embedded in Epon. Ultrathin sections were cut, placed on 200 mesh copper electron microscope grids, stained with uranyl acetate, and examined with a JEM-2000EXII transmission electron microscope.

### 2.10. Histological Evaluation

For histological evaluation, the tissues were fixed with freshly prepared 4% buffered paraformaldehyde, embedded in paraffin wax, and sectioned. Sections were stained with hematoxylin-eosin (H&E) and subsequently examined by light microscopy. Each specimen was scored using the following criteria [19]: 0 = normal lung; 0.5 = 1 or 2 foci of 10–20 cells per section, or small areas with twofold-thickened alveolar septa; 1.0 = 3–5 foci of 10–30 cells per section, or widespread areas with twofold-thickened alveolar septa; 2.0 = 5^+^ foci of 10–50 cells per section, or two- to three-fold-thickened alveolar septa throughout the lung; and 3.0 = 5^+^ foci of 10–100 cells per section, or three- to four-fold-thickened alveolar septa throughout the lung. 

### 2.11. *In Situ* Hybridization for Viral DNA

For *in situ* hybridization, lung tissue sections were permeabilized with proteinase K (100 *μ*g/mL) for 20 min, washed with PBS, and postfixed with 4% formaldehyde (from paraformaldehyde) for 10 min. The sections were then treated with 0.3 M NaOH for 5 min and neutralized with 0.4 M Tris-HCl (pH 7.4) for 15 min. The sections were subsequently washed with PBS and sequentially dehydrated with 50% and 100% ethanol. Prehybridization was carried out in buffer (50% formamide, 1× SSC, 1× Denhardt's, 500 *μ*g/mL salmon sperm DNA) at room temperature for 2-3 h. Specimens were then covered with a hybridization buffer (prehybridization buffer supplemented with a digoxigenin-conjugated oligonucleotide probe (Table 1) targeted to MCMV IE1 gene and 10% dextran sulfate) and incubated for 10 min at 85°C and overnight at 37°C. Following hybridization, slides were sequentially washed with 2× and 0.1× SSC. The sections were incubated with alkaline phosphatase-conjugated antibodies to digoxigenin (Roche Diagnostics, Mannheim, Germany) for 2 h, washed, and visualized by histochemical detection of alkaline phosphatase activity [20]. Levamisole (0.5 mM) was added to the substrate to inhibit endogenous alkaline phosphatase activity in the tissue.

### 2.12. Immunohistochemistry

Sample collection and immunohistochemical staining were performed as described previously [21]. Briefly, paraffin-embedded lung sections were deparaffinized and hydrated through graded alcohol washes. The sections were blocked with PBS supplemented with 0.2% Triton X-100 and 10% normal horse serum for 60 min at room temperature. They were then incubated with anti-CMV gB monoclonal antibodies (Virusys Corp., MD, USA) overnight at 4°C. The samples were then incubated with HRP-conjugated rabbit anti-mouse IgG (Fab fragments) for 2 h at room temperature, followed by 3,3′-diaminobenzidine. Images were obtained with a digital camera (Leica CTR 6000 microscope, Germany) and analyzed with Velocity 5.4 imaging software (Improvision, Waltham, MA, USA). 

### 2.13. Statistical Analysis

Comparison among groups was performed via analysis of variance. Mean values were compared using Student's *t*-test. *P* values of <0.05 were considered statistically significant. The statistical analyses were performed using SPSS software version 13.0 (SPSS Inc., Chicago, IL, USA).

## 3. Results

### 3.1. Transplant Performance

**Figure 1:**
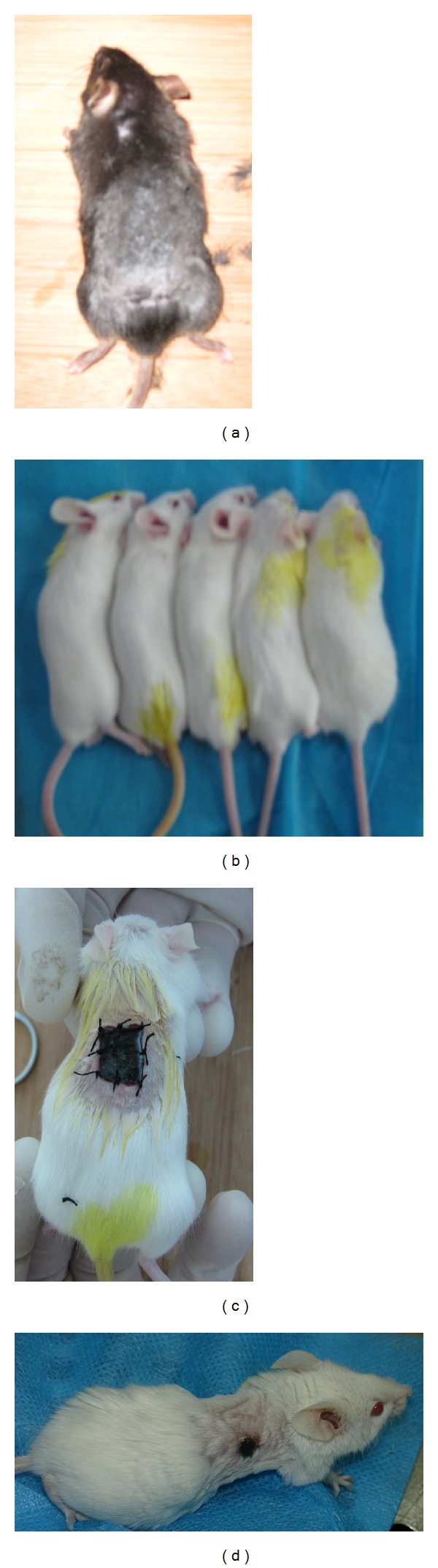
Skin transplantation from C57BL/6J mouse to BALB/c mice. (a) Shaved C57BL/6J donor mouse before transplantation. (b) BALB/c recipient mice before transplantation. (c) Recipient mouse at 0.5 d after transplantation. (d) Recipient mouse at 4 d after transplantation.

Skin grafts from 20 C57BL/6J mice were successfully transplanted to 112 BALB/c mice (Figure 1). Around 8-9 days after transplantation, skin grafts on the recipient BALB/c mice began to scab off. In the Control B and Control BV groups, most of the skin grafts showed necrosis because of graft rejection in the absence of CsA. Of the mice in experimental Groups A–E, in which CsA was administered continuously for 14 days, subcutaneous blood vessels under the skin grafts were found in 72 (90%) of the transplanted mice. Additionally, 18 (22.5%) mice showed growen hair on the skin grafts. 

### 3.2. Immune Status of Mice

In order to determine the immune status of the mice receiving CsA and transplants, the CD4^+^ and CD8^+^ cells in the peripheral blood of the animals were examined by flow cytometry, and IFN-*γ* levels in blood were assayed by ELISA. It was found that CsA, but not the allografts, decreased the numbers of both CD4^+^ and CD8^+^ cells significantly (Table 2). Similarly, IFN-*γ* levels in the CsA-treated groups (Controls A and AV) were much lower than in the untreated groups (Controls B, BV, C, and CV). All transplanted mice (Groups A–D) exhibited gradually decreasing levels of IFN-*γ* as the virus dose was increased (*P* < 0.05) (Table 3). In response to viral infection, the number of CD4^+^ cells and the CD4^+^/CD8^+^ ratio both decreased in MCMV-infected control groups (Controls AV, BV, and CV) than in the corresponding virus-free control groups (Controls A, B, and C, resp.). Also, IFN-*γ* levels in the MCMV-infected control groups (Controls AV, BV, and CV) were slightly lower than those in the corresponding control groups (Controls A, B, and C, resp.). This suggests that viral infections may decrease the level of cellular immunity. Therefore, we ensured that the transplant recipients lived in an immunosuppressed state via continuous CsA administration after grafting compared with normal mice.

### 3.3. The Physiological Changes of Recipient Mice after MCMV Infection

More pathophysiological manifestations, such as lethargy and anorexia, were observed in MCMV-infected control groups (Controls AV, BV, and CV) compared with those not treated with CMV (Controls A, B, and C). The symptoms were particularly evident in the Control AV group. In the experimental groups, lethargy and anorexia were observed in animals of Group D, but not in mice of the other groups. The difference between Group D and the other groups was significant. 

**Figure 2:**
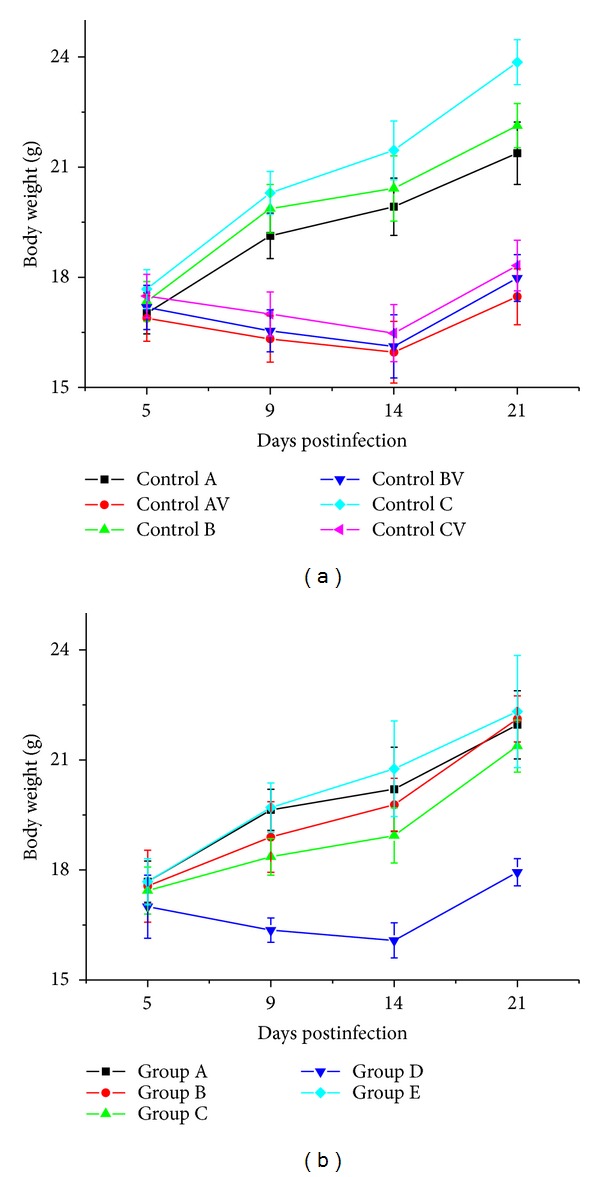
(a) The body weight of mice of the control groups (Controls A, B, C, AV, BV, and CV) at different days postinfection. (b) The body weight of mice of the experimental groups (Groups A–E) at different times after MCMV infection.

Furthermore, the growth of MCMV-infected mice (Controls AV, BV, and CV), as measured by body weight, was slower than those of the corresponding control groups (Controls A, B, and C) (Figure 2(a)) (*F* = 9.213, *P* < 0.05). The growth of mice in group D was also slower than the growth of the uninfected mice of Group E (*F* = 10.492, *P* < 0.01) (Figure 2(b)).

### 3.4. Virus Isolation

As shown in Table 4, no virus was isolated from mice of the negative control groups (Controls A, B, and C). However, from the three MCMV-infected control groups, MCMV was isolated from 43.8% (Control AV), 18.7% (Control BV), and 12.5% (Control CV) of the mice. These results imply that the use of CsA in MCMV-infected mice can promote viral proliferation. 

**Figure 3:**
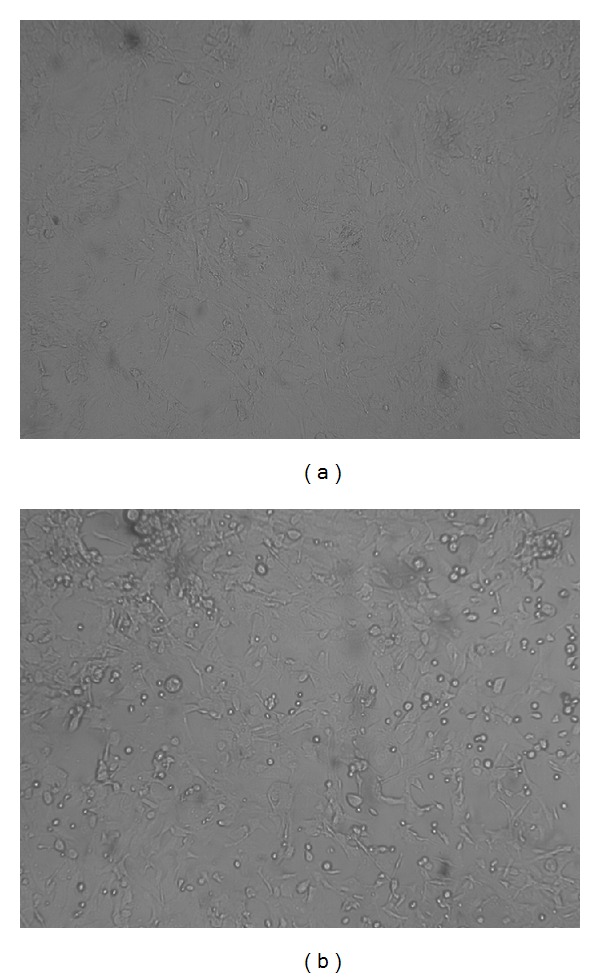
MCMV isolation from lung tissue of infected mice (unstained, ×100). MEF cells were incubated with suspension of lung tissue homogenates. (a) No CPE was present in MEF cells incubated with lung tissue homogenate from a mouse in Group E (uninfected control). (b) CPEs were observed in MEF cells incubated with lung samples from a mouse in group D.

MCMV was isolated from lung tissue of the animals of Groups C (37.5% or 6/16) and D (62.5% or 10/16) at 5, 9, and 14 days after infection (Figure 3). No virus was isolated from mice that received virus <10^3^ PFU. 

### 3.5. PCR and RT-PCR

**Figure 4:**
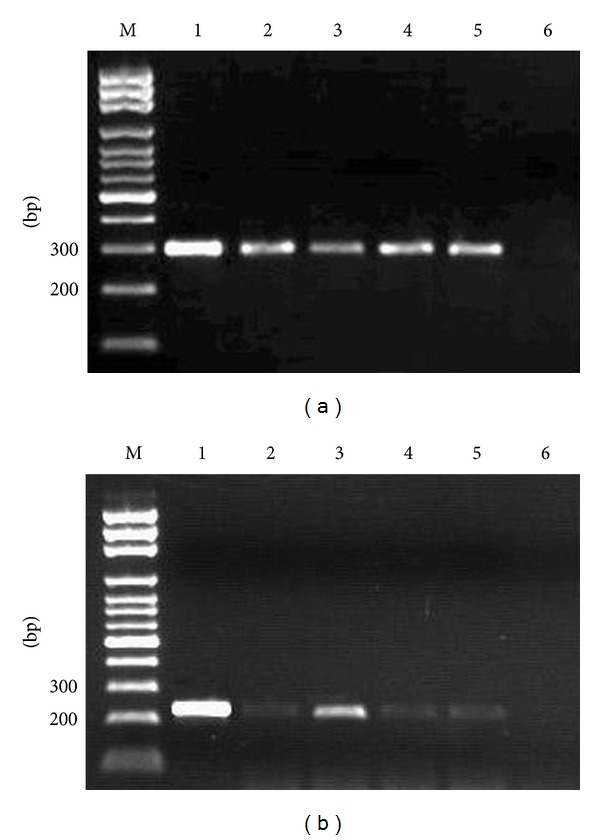
(a) MCMV IE gene expression in lung tissue as determined by RT-PCR assay. M: DNA marker. 1: positive control. 2–5: IE gene expression in mice of Group D at 5, 9, 14, and 21 days postinfection. 6: IE gene expression in mice of group E (negative control). (b) MCMV M55 DNA in lung tissue as determined by RT-PCR assay. M: DNA marker. 1: positive control. 2–5: mice of Group D after being infected with MCMV 5, 9, 14, and 21 days. 6: Sample from mice in Group E (negative control).

The results of PCR and RT-PCR analyses are shown in Table 4. No PCR and RT-PCR products were obtained from the negative control groups (Controls A, B, and C). However, in the MCMV-infected control groups, MCMV IE and M55 DNA were successfully amplified from each group's tissue (100%). The transcript from the MCMV IE gene was detected in 100% of the animals. The transcript from the MCMV M55 gene was found in 25%–50%. These results suggest that the application of CsA was able to enhance the replication and propagation of MCMV in host mice. In the experimental groups, PCR and RT-PCR studies revealed that MCMV IE-1 DNA and MCMV IE-1 RNA appeared in lung tissues as early as 5 days postinfection (Figure 4(a)). In addition, MCMV late gene M55 transcripts were detected by RT-PCR between 5 and 21 days postinfection (Figure 4(b)). These results suggest that productive infection occurred in the lung tissue of mice treated with MCMV.

### 3.6. Real-Time PCR

**Figure 5:**
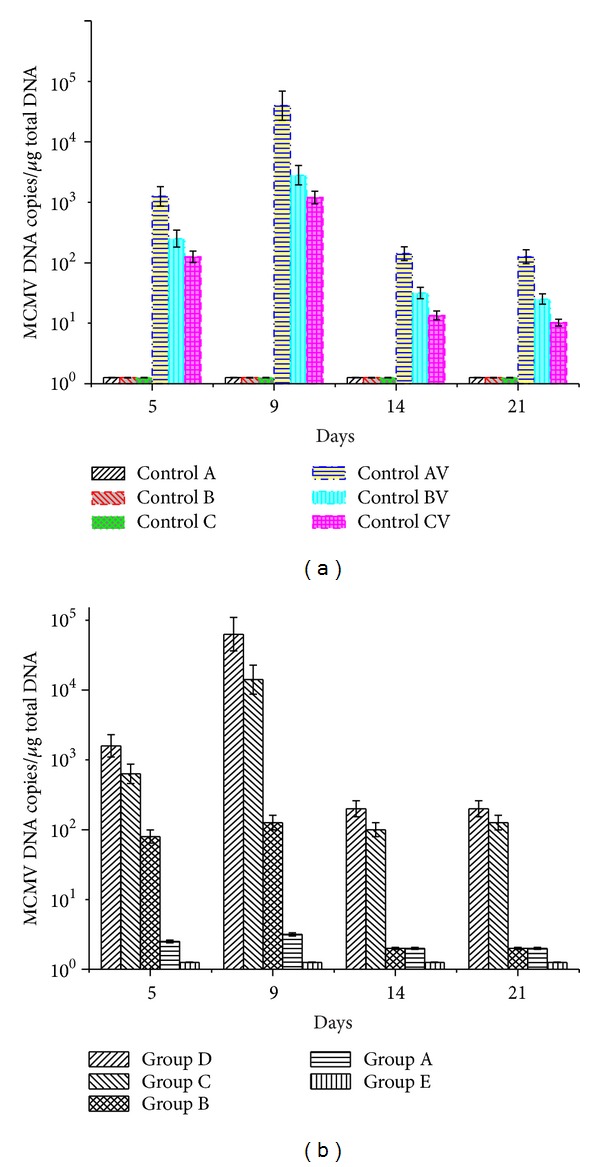
(a) MCMV viral load in lung tissues of BALB/c mice of the control groups (Controls A, B, C, AV, BV, and CV) at 5, 9, 14, and 21 days postinfection. The DNA copy numbers were determined by real-time PCR assay (*n* = 3 at each time point per group). (b) MCMV viral load in the lungs of BALB/c mice of the experimental groups (Groups A–E) at 5, 9, 14, and 21 days postinfection. The DNA copy numbers were determined by real-time PCR assay (*n* = 3 at each time point per group).

MCMV DNA copy numbers in the lung tissues of recipient mice were analyzed by real-time PCR. The average viral copy number increased between 5 and 9 days postinfection and then decreased 14 and 21 days postinfection (Figure 5). The results were consistent with the previous reports of acute MCMV infection in adult mice after 3-4 weeks of effective infection [22]. For the three negative control groups (Controls A, B, and C), the DNA copy numbers of MCMV were near to zero. The MCMV DNA copy number was higher for Group AV compared with that of Control BV and Control CV (Figure 5(a)). Among the five experimental groups (Groups A–E), the highest MCMV DNA copy number appeared in Group D (5.6 × 10^4^ copies/*μ*g) at 9 days postinfection (Figure 5(b)). This fits the cut-off value of CMV-IP reported previously [23]. 

### 3.7. Transmission Electronic Microscopy (TEM)

**Figure 6:**
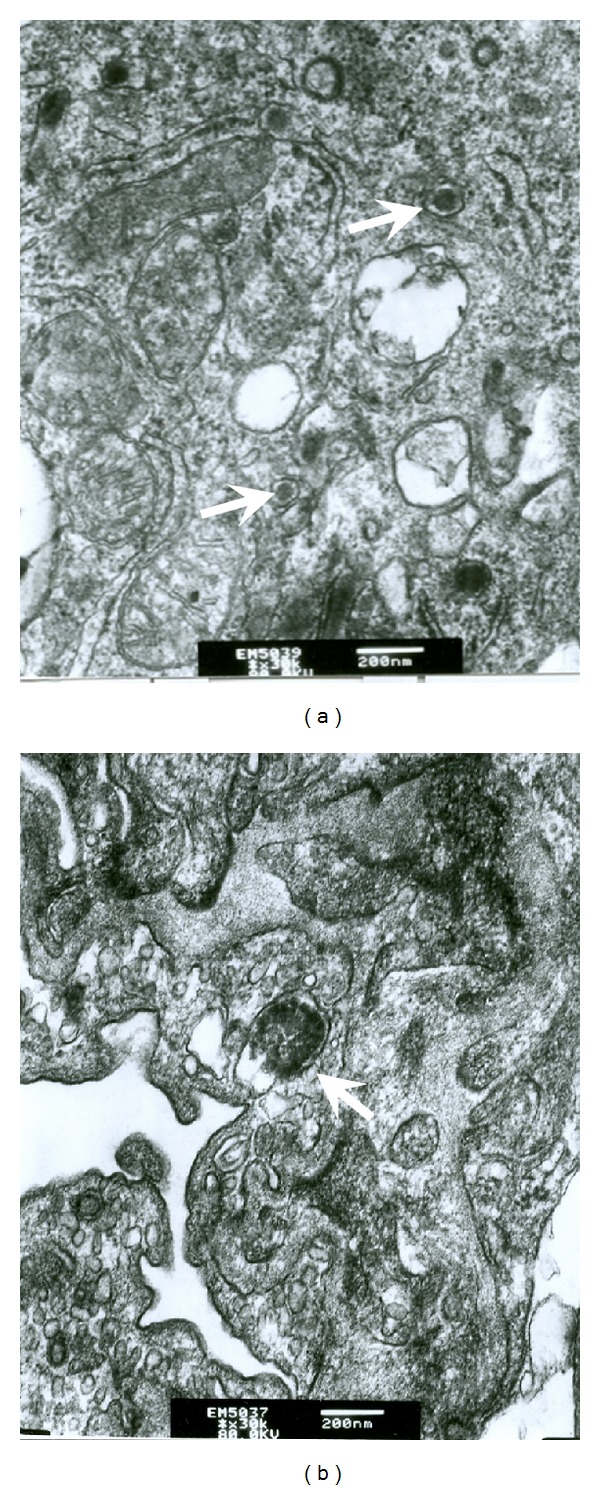
Herpes-like virus particles in the alveoli of lung tissues as observed by TEM. (a) Herpes virus-like nucleocapsids (arrows) were present in an epithelial cell of the alveoli of a mouse of Group C 21 days postinfection (×50,000). (b) An inclusion body was found in the cytoplasm of an epithelial cell of the alveoli of a mouse in Group D 21 days postinfection (×30,000).

TEM was used to visualize the viral particles of MCMV in the lung tissue of infected mice. Herpes-like virus particles and inclusion bodies were imaged by TEM in the epithelium of the lung tissue in mice of Control AV, Control BV, Control CV, Group C, and Group D (Figure 6). None of the virus particles or inclusion bodies were found by TEM in the lung tissue of other mice groups.

### 3.8. H&E Staining

**Figure 7:**
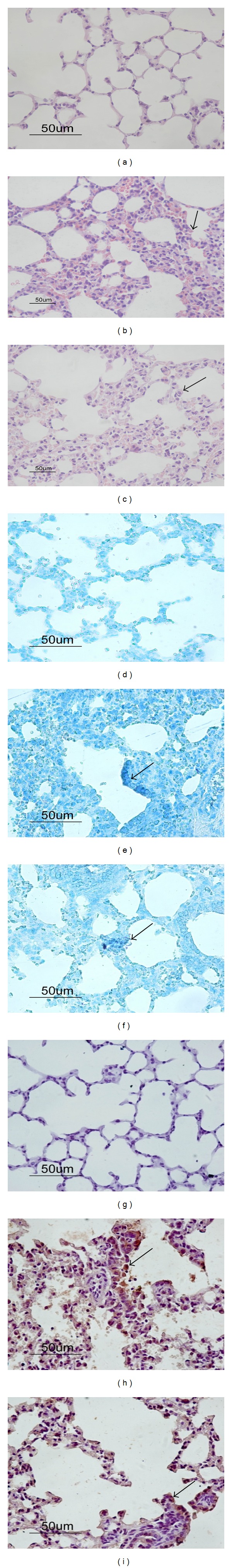
(a)–(c) H&E staining of lung tissue from infected or mock-infected mice. (a) Tissue from Group E (the mock-infected group) showed the normal histology of the mouse lung. (b)-(c) Tissues from Group D (b) 14 or (c) 21 days postinfection show mononuclear cell infiltration and edema of alveolar epithelia (arrows). (d)–(f) *In situ* hybridization with MCMV-specific probes showed that MCMV DNA was present in epithelial cells (arrows) of the alveoli in the lung tissue of mice (e) 14 or (f) 21 days postinfection, but not in (d) mock-infected mice. (g)–(i) Immunohistochemical staining using anti-MCMV gB monoclonal antibodies revealed viral protein gB (yellow-brown color, indicated by arrows) appeared in the alveolar epithelia (h) 14 and (i) 21 days postinfection. (g) We observed no staining in the lung tissue of mock-infected mice. Bar = 50 *μ*m.

In order to confirm that the mouse model was a useful model of MCMV interstitial pneumonia, histological analysis was carried out on tissues from infected and mock-infected mice. Lung tissues from mice of the MCMV-infected control groups (Controls AV, BV, and CV) were markedly impaired, as evidenced by the dense inflammatory foci, compared with those from the negative control groups (Controls A, B, and C). The same pathological appearance was observed in Group C and Group D, which was different compared with Group E. The most severe abnormality appeared 14 days after infection. The inflammatory foci diffused through the lung parenchyma. The walls of the pulmonary alveoli had thickened, likely due to the edema of alveolar epithelia, proliferation of interstitial cells and interstitial lymphocytes, and inflammatory infiltration of mononuclear cells. In addition, the alveolar space became smaller, and the compensatory emphysema was found on the lobe edges. No abnormalities were noted in the lungs of uninfected Group E mice. Histological scoring indicated that the lungs of infected mice had significant interstitial inflammation (Figure 7).

### 3.9. *In Situ* Hybridization

Digoxigenin-labeled, MCMV IE-specific probes were used for the detection of MCMV DNA in lung sections of mice from the experimental and control groups. Blue-violet-stained cells were found in the lung epithelia and lung interval of mice of the MCMV-infected control groups (Control AV, Control BV, and Control CV) 21 days postinfection. Among the experimental groups, similar results were found in Group C and Group D at 14 and 21 days postinfection. On the contrary, no positive signal was observed in the mock-infected mice (Control A, Control B, Control C, and Group E). 

### 3.10. Immunohistochemistry

No MCMV antigen was detected in lung tissues from mice of the mock-infected groups (Control A, Control B, Control C, and Group E). However, in the MCMV-infected control groups (Control AV, Control BV, and Control CV) and Groups C and D, MCMV gB was found in lung epithelial cells and endothelial cells at 14 days postinfection, as well as in the interstitial alveolar epithelial cells at 21 days postinfection by immunohistochemistry (Figure 7). No MCMV gB antigen was detected in the lung tissue of mice of Group A and Group B.

## 4. Discussion

 Traditionally, research systems studying the molecular biology and pathogenic mechanisms of CMV use either human cells cultured with human CMV *in vitro* [24] or animal CMVs in their natural host species [25]. Compared with those *in vitro* studies, *in vivo* approaches have advantages because they resemble more closely the complexity of human conditions.

For HCMV, a few models of HCMV infection were developed in which human fetal materials were implanted into laboratory animals to support viral infection *in vivo*. Allen et al. [26] used HCMV-infected MRC-5 cells entrapped in agarose plugs before implanting them intraperitoneally into mice. 

A mouse model of severe combined immunodeficiency disease (SCID) [27] was developed in which conjoint implants of human fetal thymus and liver were placed under the kidney capsule with subsequent infection of HCMV [28]. In another model, fragments of human fetal retina were implanted in the anterior chamber of the eye in athymic rats to support HCMV growth [29]. Immunocompetent animal models of HCMV infection were also developed by direct injection of the virus. Dunkel et al. [30] established an animal model of progressive HCMV chorioretinal disease by injection of HCMV (10^5^ PFU) into the rabbit vitreous. Tang et al. developed a mouse model hallmarking the congenital human cytomegalovirus infection in the central nervous system and showed that HCMV could vertically transmit through the placenta of mice to infect their offspring in the central nervous system [20].

For MCMV, animal models of MCMV infection were developed to induce interstitial pneumonia in untransplanted or transplanted mice. Jordan [31] established a murine model by intranasal administration of MCMV. They showed that MCMV could be the sole pathogen responsible for severe interstitial pneumonitis in normal mice when the mice were given greater than or equal to 10^4^ PFU of MCMV intranasally. Fitzgerald et al. [32] described a newborn mouse model of MCMV infection to examine the pathogenesis of MCMV infection in resistant and susceptible mice on the day of birth. It was found that BALB/c mice developed severe interstitial pneumonitis 10 days postinfection. 

Because HCMV may result in severe CMV-IP after autologous bone marrow transplantation (BMT) [33], Podlech et al. [34] used a model of syngeneic BMT and simultaneous infection of BALB/c mice with MCMV to study the pathogenesis of CMV-IP by controlled longitudinal analysis. On the contrary, Yonemitsu [15] constructed a model of latent MCMV infection showing that MCMV alone could not induce the development of interstitial pneumonia without the help of IL-4 in latent MCMV-infected mice. Therefore, IL-4 appears to be a key cytokine for the onset of interstitial pneumonia in mice with latent MCMV infection. 

Because a mouse model of MCMV interstitial pneumonia with allogeneic skin transplantation was not fully described, we tried to develop a progressive mouse model of CMV pneumonitis following skin transplantation.

 Firstly, we performed the skin transplantation between C57BL/6J and BALB/c mice and administrated CsA to suppress allograft rejection. While most of the recipient mice exhibited subcutaneous vascular formation and hair growth in skin grafts after 14 days, about 13% of the transplanted mice showed reduced physical activities and weight loss. Flow cytometry showed that the number of CD4^+^ and CD8^+^ cells and the level of IFN-*γ* decreased significantly in peripheral blood of the recipient mice compared with those of normal mice (*P* < 0.05). This suggested that those recipient mice were under immunosuppression status.

Secondly, we infected the mice with MCMV by intranasal administration. Five, 9, 14, and 21 days postinfection, lethargy, anorexia, and weight loss were observed in the mice of Group D, which received a high dose (10^5^ PFU) of virus. We did not observe these traits in the mock-infected group of mice and those that received lower doses of virus. Pathological examination of the lung tissue demonstrated mononuclear cell infiltration, alveolar hemorrhage, and interstitial lung cell enlargement. The walls of the pulmonary alveoli thickened, probably due to the edema of alveolar epithelia and the proliferation of interstitial cells. Those pathologic features were less apparent in the animals that received lower doses of virus. These observations demonstrated that the progressive course of acute interstitial pneumonitis occurred after MCMV infection of skin-transplanted mice, and the replication of virus in lung tissues resulted in pathological abnormalities.

Mice of Controls AV, BV, and CV may develop interstitial pneumonitis, suggesting that MCMV infection is the major cause of interstitial pneumonitis. CsA in these groups aggravated the inflammatory response and promoted virus replication. Infection with the highest dose of MCMV in Group D resulted in the most severe lung pathological features and interstitial pneumonitis-like pathological changes at 14 d and 21 d postinfection. These results implied that a certain quantity of virus (e.g., 1 × 10^5^ PFU in this study) was needed to effectively produce typical interstitial inflammation.

On the other hand, mice of the negative control groups (Controls A, B, and C) did not develop interstitial pneumonitis, which suggests that the use of both allograft and CsA or any of them individually could not induce interstitial pneumonitis in mice.

 Our study showed that successful MCMV replication was due to a combination of skin transplantation and CsA immunosuppression. Immunological injury, immune activation, and immunosuppression may be involved in virus activation and replication, the mechanisms of which need further research. Although this model involved a large number of allogeneic animals, the method is relatively easy, simple, and economical. Features of this newly developed animal model are mimicked with HCMV infection after solid organ transplantation in humans [3]. This model, therefore, is useful for further study of the roles of HCMV infection in the pathogenesis of interstitial pneumonia in transplant recipients. 

In conclusion, we described here a mouse model of MCMV-induced interstitial pneumonitis after skin transplantation. MCMV replication is responsible for the progression of pneumonitis. Thus, this model would enhance the understanding of the pathophysiology of MCMV infections and could be useful in studying the prevention of and interventions for HCMV pneumonitis or pneumonia in patients receiving transplants.

## Figures and Tables

**Table 1:** Primers and probes for PCR/RT-PCT, real-time PCR, and *in situ* hybridization.

Primer name	Primer or probe sequences	PCR product size and reference
IEA Dig probe	5′-TTCTCTGTCAGCTAGCCAATGATATCTTCGAGC-3′	[17]

IEA primers	Forward: 5′-TACAGGACAACAGAACGCTC-3′ Reverse: 5′-CCTCGAGTCTGGAACCGAAA-3′	300 bp [17]

M55 primers	Forward: 5′-GCGACATACACTTCTCCATT-3′, Reverse: 5′-CAGAATACGTGGCTCACA-3′,	209 bp

IEB primers and probe	Forward: 5′-TGCCATACTGCCAGCTGAGA-3′ Reverse: 5′-GGCTTCATGATCCACCCTGTT-3′ Probe (FAM): 5′-CTGGCATCCAGGAAAGGCTTGGTG-3′	66 bp [18]

Mouse *β*-actin primers and probe	Forward: 5′-GAAGATCAAGATCATTGCTCCT-3′ Reverse: 5′-TACTCCTGCTTGCTGATCCA-3′ Probe (FAM): 5′-CTGTCCACCTTCCAGCAGA-3′	111 bp [18]

**Table 2:** T lymphocyte subtypes in mouse peripheral blood.

Mouse group	The proportion of T lymphocyte subtypes		
	($\overset{-}{x}$% ± SD, *n* = 4)		
	CD4^+^	CD8^+^	CD4^+^/CD8^+^
Control A	16.28 ± 1.29	11.46 ± 0.51	1.42 ± 0.30
Control B	25.78 ± 1.52	14.82 ± 0.60	1.74 ± 0.52
Control C	23.70 ± 1.43	14.40 ± 0.65	1.65 ± 0.51
Control AV	14.03 ± 1.30	10.79 ± 0.48	1.30 ± 0.39
Control BV	23.28 ± 1.41	14.93 ± 0.62	1.56 ± 0.45
Control CV	21.75 ± 1.48	14.50 ± 0.59	1.50 ± 0.47
Group A	17.74 ± 1.41	12.32 ± 0.58	1.44 ± 0.40
Group B	17.46 ± 1.36	12.38 ± 0.54	1.41 ± 0.32
Group C	17.07 ± 1.33	12.46 ± 0.49	1.37 ± 0.23
Group D	16.42 ± 1.32	12.53 ± 0.51	1.31 ± 0.22
Group E	18.20 ± 1.69	12.24 ± 0.52	1.49 ± 0.24

**Table 3:** IFN-*γ* levels in mice plasma.

Mouse group	Mean ± SD (pg/mL)
Control A	22.875 ± 0.682
Control B	26.897 ± 0.874
Control C	26.073 ± 0.921
Control AV	20.674 ± 0.645
Control BV	24.382 ± 0.674
Control CV	24.012 ± 0.753
Group A	20.442 ± 0.482
Group B	19.845 ± 0.490
Group C	19.027 ± 0.478
Group D	18.124 ± 0.471
Group E	22.753 ± 0.507

**Table 4:** Detection of MCMV or its DNA or RNA in lung tissues from mice of all groups.

Group	Number	Virus isolation (%)	Viral DNA (%)		Viral mRNA (%)	
			MCMV IE	MCMV M55	MCMV IE	MCMV M55
Control A	16	0	0	0	0	0
Control B	16	0	0	0	0	0
Control C	16	0	0	0	0	0
Control AV	16	43.8	100	100	100	50
Control BV	16	18.7	100	100	100	31.3
Control CV	16	12.5	100	100	100	25
Group A	16	0	100	100	100	0
Group B	16	0	100	100	100	43.8
Group C	16	37.5	100	100	100	62.5
Group D	16	62.5	100	100	100	75
Group E	16	0	0	0	0	0
